# Mineral and Thermal Springs of the United States

**Published:** 1855-07

**Authors:** John Bell


### *Mineral and Thermal Springs of the United States.*

By John Bell, M.D.

(Concluded.)

*Warm Springs.*—They are situated in a valley in the county of Bath, between two ranges of mountains, 170 miles from Richmond and 50 from Staunton, on the turnpike road which leads to the Ohio. There is not a more delightful natural warm bath to be found in any part of the world than is obtained in the reservoir which receives the water of the chief spring. Its temperature at first 98° F., is soon reduced to 96°, and for mere luxurious enjoyment it would merit a visit from Hudson's Bay, not to speak of Canada or New England. The large bath is of an octagonal form, 38 feet in diameter, and, on an average, five feet deep. Nitrogen gas is largely evolved, and, in small quantities, carbonic acid and sulphuretted hydrogen gases. The solid contents are in small quantity, not 24 grains in a gallon of the water. In addition to the large octagonal bath for gentlemen, there are now a ladies' bath and a spout bath, and also a cold plunging bath near the first of these. The quantity of water given out by the warm spring has been estimated to be a thousand gallons a minute.

All that has been said of the enjoyment from warm bathing, and its utility in a long list of diseases, is applicable to the external use of the Warm Spring water. Having already enlarged on this subject in my work on "Baths and the Water Regimen," I shall not dwell on it at this time. Adequate attention is not paid to the systematic use, internally, of this water, which, although possessed of little mineral strength, might be made a powerful and an efficacious auxiliary to the bath. In plethoric and inflammatory states of the system, and with a tendency to cerebral determination and excitement, caution must be displayed in the large or prolonged employment of this double remedy; for, although the temperature is such as to bring it within the limits of the warm bath, it comes so near the hot as to prove in many cases a stimulant, and, as such, it is not to be trifled with under the circumstances just mentioned.

The house at the Springs is one of the best.

*Hot Springs.*—These Springs are situated five miles west of the Warm, and in the same county of Bath. The hotels and cabins have little in their appearance to recommend them, but the table is said to be very good. The water of the baths—six in number—is from 98° to 106° F., and it is so distributed as to allow of its use by immersion in the common way, and by the douche or spout. As no person in his senses, who is in common health, ought to take a *hot* bath for mere pleasure, these waters are resorted to by the sick and invalid, and hence there is not the crowd at the Hot which is so common at the other springs, especially the White Sulphur.

Unable to enter into minuteness of detail as to the modes and times of employing the waters, or to specify the diseases to which they are applicable, I must content myself with remarking that they are too powerful to be had recourse to except under medical guidance. As excitants of the first class, they are only adapted to diseases of functional debility, without inflammation or active congestion, or fever. In chronic rheumatism and gout, and in chronic stomachic and intestinal diseases, in which the circulation is languid and the skin cold or clammy, the tongue moist, with an absence of thirst, the hot bath and hot douche and drinking the hot water display, often, wonderfully restorative powers. So, also, in tumid livers and spleens after a subsidence of fever and phlogosis, in paralysis where the brain has recovered its functions, and in stiff and anchylosed joints and indolent and scrofulous tumors, old ulcers and chronic diseases of the skin, especially of the scaly kind, these means deserve a full trial. The application of hot water by douching adds greatly to its power.

*Healing Springs.*—These springs, only noticed within a few years past, are three and a half miles south of the Hot Springs. The temperature of the water, as tested by Dr. Burke, is 84° F. In composition, he represents them to resemble closely the Sweet Springs, and perhaps the Red Sweet more. They are, according to the popular belief of the neighborhood, invested with miraculous powers. They are most extolled in rheumatism, sprains, herpetic eruptions, and scrofulous ulcers. As a bath their temperature makes them adapted to a large class of persons.

*The Group of Sulphur Springs.*—On leaving the Hot Springs the traveller reaches, after a journey of 35 miles in a southwestern direction, the White Sulphur Springs; and if he continue on westwardly he comes to the Blue Sulphur, distant 22 miles from the White. The Salt Sulphur Springs are 24 miles southwest from the White, and the Red Sulphur are 17 miles further on in the same direction. This is not the place to speak of the geological relations of these several Springs, nor to inquire how far they are all referable to the class of what have been called, of late, by some, Secondary, or Accidental Sulphureous Springs, in contrast with those of the Pyrenees, chiefly thermal, by the way, which alone are thought to belong to the class of the Natural or Primary Sulphureous. For the latter have been claimed a therapeutic energy by no means proportionate to the amount of their constituent principles; so that some of them, with only half or even the fourth of the latter contained in the secondary class, will, we are told, display much more curative power. These are points on which I can only enlarge with advantage in my forthcoming volume,* in which the natural history of both common and mineral, and thermal springs, and their peculiar features, as exhibited in different parts of the world, will be given in requisite detail.

Whatever result may be reached in an inquiry of this kind, no one can contest the really curative virtues of the waters of this entire group; at the same time, no one will deny that they are all endowed with such therapeutical energy that they must be either beneficial or mischievous, according to the wisdom of the prescriber in adapting their use to meet the requisite indications. No visitor at a sulphur spring can gorge himself with the waters, for experiment or amusement, with impunity. In fact, few remedies are as diffusive in their action on the animal frame, or as searching and alterative on the tissues, as the sulphureous. But, in order to produce their full and salutary effects, they ought to be administered in quantities, or doses, of moderate strength and for a lengthened period, and in that state of dilution in which they are found in mineral springs. By this means we shall avoid, in a great measure, that excitement and disturbance of function, resulting from the common indiscriminate use of the waters, which not seldom constitutes a disease in itself, or brings back with aggravation the original malady they were intended to remove. Some, particularly the German physicians, insist on the necessity of a morbid reaction of this kind, especially after the prolonged use of baths of a high temperature, which they call bad-storm or commotion. Were we to admit this, it ought to be regarded as a conclusive reason for a longer time being taken and greater patience displayed by the invalid, and more watchful superintendence on the part of the physician than are conceded for the treatment of a case of chronic disease at any of the sulphur springs. To do full, we ought rather to say common justice to such a case, the methodical and

* I do not refer now to the Manual on the Mineral and Thermal Springs of the United States which is now in press; but to a more complete work in progress, of which this is but an instalment.

regular use of the bath must be enjoined with drinking of the water.

Experience has pretty well established the fact of the water of the *White Sulphur Springs* on Howard's Creek, the original White Sulphur, of Greenbriar county, being the strongest, most active and stimulating, and, therefore, when misapplied, the most mischievous of the group; and which requires the greatest caution in its use. These matters will be regulated by physicians on the spot. Among these, Dr. Moorman has given us the result of his now somewhat long experience of the remedial value of the White Sulphur Water, in a small volume, entitled "The Virginia Springs." The temperature of this water, 57°, (he says 62° F.) is some degrees higher than that of the common springs of the district, and makes it rank in the quasi thermal class.

The *Red Sulphur Spring* furnishes a water which is the mildest of the group, and perhaps of its class, and it has even been regarded by some as sedative in its operation on the animal economy, although this is a contested point. Still more, it has been extolled for its power to cure pulmonary consumption itself. Instances are recorded of its effects on the action of the heart, so as to reduce the beats of this organ from upwards of 100, and even 120 and 130, to 70 and 65 in a minute. Drinking of it allays thirst and causes sleep. That it has a really soothing and salutary effect in tracheal and bronchial irritation seems to be pretty evident; but of its curing consumption we have, I believe, no well authenticated proof. It will not probably aggravate this disease, as the water of the White Sulphur does. For details of the operation and curative powers of the Red Sulphur, the reader is referred to the work of Dr. Burke, on "The Mineral Springs of Virginia."

*Salt Sulphur Springs.*—The designation of salt is hardly explicable by the very minute quantity of chloride of sodium which enters into the composition of these waters. It contains larger proportions of sulphates of soda and magnesia. The temperature varies from 49° to 56° F. As more aperient and diuretic than some others of its class, the Salt Sulphur, though it must still be ranked as an excitant, is applicable to mixt cases of febricula and languor, as in chronic dyspepsia and renal affections, and chronic diarrhœa; in some of which the frequency of the pulse has been diminished under its use. The water of the "New Spring," with a smaller proportion of saline matters, has more evident traces of iodine than the "Old Spring."

*The Blue Sulphur Springs* (Greenbriar county) are thirty two miles from the Red Sulphur, and nearly the same distance from the Salt Sulphur Springs, and twenty-two miles from the Sweet. Resembling in their chemical properties those of the White Sulphur, these waters are applicable to the same diseases, and require the same precautions for their use as the former.

*The Sweet Springs*, among the first of the mineral springs of this region which were visited by invalids, still retain their early attractions. They are situated on the eastern border of Monroe county, seventeen miles from the White Sulphur and twenty-two from the Salt Sulphur, in a beautiful valley, bounded on the north by the Alleghany, and on the south by the Sweet Springs mountain. It is the spot where the last rally of visitors is made for the season, before separating for their several homes.

The temperature of the water, 74° F., places it in the thermal class. In taste sub-acid and slightly alkaline, and evolving freely carbonic acid, it belongs to the class of acidulous waters, and may be used with the same benefit as these are. It is applicable, therefore, to irritable dyspepsia, with gastralgia, and to renal and hepatic colic, and to bilious diarrhœa. In renal affections, and especially in those of the lithic and phosphatic diathesis, and in irritable bladder, it is calculated to do much good, when continued for an adequate period. The same remark applies to chronic gout, of which these disorders are often varieties.

The full therapeutical value of the water of the Sweet Springs is far from being properly appreciated; and if a careful clinical record of each disease regularly subjected to its use were to be kept for a few seasons, by a resident physician, it would show, that these springs might be compared most advantageously with some of the most noted of the class on the continent of Europe. Their tendency, after a time, to produce constipation, and their immediate effects, evinced in fulness of the head and often drowsiness, point to their cautious use, if not, for a while, at least, their being withheld, in general and also in local plethora.

The copious supply of the water for bathing, and its temperature, are such as to attract many to the spot on this account alone, who would not take a cold bath, but who find in this temperate one a means of refreshment and invigoration, some would say rejuvenation, in which old Jason himself might have rejoiced, notwithstanding the marked preference of his daughter and doctress Medea for the hot bath.

*Red Sweet Springs.*—These are only a mile distant from the Sweet Springs, which they closely resemble in all essential particulars; with the exception of their being more evidently impregnated with iron than the latter. The two prominent gases are nitrogen and carbonic acid, in the proportion, respectively, of 62.5 and 37.5 per cent. Of the three springs, the upper has a temperature of 77° F., the middle 80°, and the lower 79°, the waters of which blended into one stream, give a temperature of about 78° F.

*Dibrell's Spring*, on the road from the Natural Bridge to the White Sulphur Springs, belongs to the sulphureous class.

*Rawley's Spring*, a strong and simple chalybeate, is in Buckingham Co., twelve miles from Harrisburg, and one hundred and twenty northeast from the White Sulphur.

*The Holstein Springs* are in Scott Co., in the southwestern angle of the State, near the Tennessee line, forty miles from Abington, and sixty from the Warm Springs of Buncome Co., N. C. One of them comes within the thermal limits, being 68°.5 F., or fifteen or sixteen degrees higher than the common springs of the country around. Of the saline constituents of the water, the chief ones are sulphates of magnesia and lime; the saline contents altogether being 41.14 grains in the gallon. It is represented by Dr. Gaines, to whom I am indebted for all the information I have on the subject, to be actively diuretic, and with suitable appliances diaphoretic. Its action on the bowels is induced, by restoring to the digestive canal its lost tone and healthy secretions. With proper caution, the bath will be found serviceable in certain cases.

In Eastern Virginia I have to speak of two springs which merit attention.

*Church Hill Alum Spring.*—This is a recent addition to the mineral springs of Virginia, having been discovered or rather opened only a few years ago, in the process of levelling a street, which bordered on the garden of a lady in the city of Richmond. The supply of water is abundant, and its mineral constituents place it nearly at the head of this class of springs. An analysis by Professor Booth shows it to contain 184.5 grains of alkaline salts, 159.5 of salts of iron, and 7.3 of persulphate of alumina in a gallon. Of the alkaline salts nearly one half is Epsom salts. On this account it is generally aperient, while, at the same time, in view of its large aluminous and chalybeate impregnation, it manifests actively tonic and astringent properties. This alum water is of great value in a number of diseases, such as passive hemmorhage, the profluvia, nervous diseases, and cutaneous and ulcerative affections. It is used to a considerable extent in Philadelphia, and I have myself often prescribed it with decided benefit. It, like others of its class, varies in its strength according as a rainy or a dry season prevailed.

*Huguenot* (formerly Howard's) *Springs.*—These are two in number, a *Sulphureous*, and a *Chalybeate*, situated near James River, in Powhattan Co., 17 miles above Richmond, on the the main river road, between that city and Lynchburg. The Sulphur Spring resembles the White Sulphur, but its water is not so strong as that of the latter. The temperature of this water is represented by Dr. Royster to be several degrees higher than any other sulphur water with which he is acquainted. The precise degree is not mentioned. Suitable arrangements have been made for the reception and accommodation of visitors at the Springs.

Here I must cease from any further attempt at even brief descriptions, being compelled to close this article for want of space. I can only allude, therefore, to the thermal springs of Buncome county, N. C., with a temperature ranging from 94° to 104° F., and those of Ouachita, (Washitaw) in Arkansas, with their vapor baths, from 90° to 154° F. Kentucky can boast of the active Blue Lick Sulphureous and Harodsburg Saline Springs; Alabama of the *Bladon Acidulous Springs*, with some sulphureous impregnation ; and *Baily's Spring, acidulous* and *chalybeate ;* also several other mineral artesian wells, obtained when boring for common water. *Cooper's Well*, in Hinds Co., and the *Ocean Springs* in Jackson Co. Mississippi, whose waters are *Sulphureous*, *Saline*, and *Chalybeate*, also merit a distinct notice. I have tolerably full notes of the mineral springs of Canada, and of the Thermal, Saline and Acidulous of Desorta, New Mexico, and California.

In conclusion, I would again renew my request to my medical brethren and other friends of science in the United States, to favor me with any information on Mineral and Thermal Springs, and also noted Common Springs which are much resorted to—in addition to those noticed in this article—so that my work may be as full and comprehensive as possible. Of the numerous acidulous and other springs of Florida I have as yet but little knowledge. Farther details respecting those of California and the whole northwestern territory would, also, be very acceptable.

